# Application of Circular Bubble Plume Diffusers to Restore Water Quality in a Sub-Deep Reservoir

**DOI:** 10.3390/ijerph14111298

**Published:** 2017-10-26

**Authors:** Chen Lan, Jingan Chen, Jingfu Wang, Jianyang Guo, Jia Yu, Pingping Yu, Haiquan Yang, Yong Liu

**Affiliations:** 1 State Key Laboratory of Environmental Geochemistry, Institute of Geochemistry, Chinese Academy of Sciences, Guiyang 550081, China; lanchen@vip.skleg.cn (C.L.); wangjingfu@vip.gyig.ac.cn (J.W.); guojianyang@vip.gyig.ac.cn (J.G.); yujia@vip.skleg.cn (J.Y.); yanghaiquan@vip.skleg.cn (H.Y.); liuyong@vip.skleg.cn (Y.L.); 2 College of Resources and Environment, University of Chinese Academy of Sciences, Beijing 100049, China; 3 College of Resource and Environmental Engineering, Guizhou University, Guiyang 550025, China; yupingpingypp@126.com

**Keywords:** bubble plume diffusers, sub-deep water system, dissolved oxygen, water quality, consumption fate of oxygen

## Abstract

Circular bubble plume diffusers have been confirmed as an effective technology for the restoration of the deep water system, but have never been applied in sub-deep water system. In this study, circular bubble plume diffusers were used, for the first time, to restore water quality in the Aha Reservoir, a typical sub-deep reservoir in Southwest China. Axisymmetric intrusive gravity currents were formed with a horizontal radius of 250 m at the equilibrium depth and the intrusion of oxygen-enriched water occurred within the depth of 10–14 m, while thermal stratification remained intact. A total of 95% of the imported oxygen was dissolved, but most was consumed by organic matter and other reduced substances within the hypolimnion. The oxygen consumption of organic matter, NH_4_^+^ and remaining reduced materials, accounted for 41.4–52.5%, 25% and 13.3–24.4% of the total imported oxygen, respectively. Compared with the control sites, dissolved oxygen level in the hypolimnion increased 3–4 times, and concentrations of NH_4_^+^, total Fe and total Mn were reduced by 15.5%, 45.5% and 48.9%, respectively. A significant decrease in total phosphorus and nitrogen concentrations was observed in the experimental zone (0.04–0.02 mg/L and 1.9–1.7 mg/L, respectively). This indicates that circular bubble plumes have great potential for oxygenation of the hypolimnion and improving water quality in the sub-deep water system. Nevertheless, further efforts are needed to improve the discrete bubble model to elaborate the oxygen transmission dynamics and the plume formation processes in sub-deep water systems, incorporating oxygen consumption processes.

## 1. Introduction

Deep water is usually anoxic (dissolved oxygen, DO < 2 mg/L), because of the consumption of oxygen with the degradation of organic matter (OM) in benthic water [1], especially when oxygen transfer is cut off by the formation of a thermocline in deep water systems [2,3,4]. The anoxic conditions may increase internal phosphorus loading and elevate primary production, which could deteriorate water quality and endanger the drinking water supply [5,6,7]. As the main countermeasure to this problem, oxygen replenishment has many advantages: (1) restraining the release of phosphorus, iron and manganese from sediment [8,9,10,11,12,13]; (2) reducing the nitrogen level by stimulating bacterial growth [14]; (3) providing favorable living conditions for benthic organisms [15]; and (4) reducing the cost of drinking water supply, and mitigating secondary pollution by disinfecting by-products [6].

The damming of rivers is one of the most widespread anthropogenic impacts on the natural environment [16,17]. There are more than 50,000 large dams (>15 m in height) in the world [18,19], roughly half of which are located in China [20]. Southwest China hosts a large number of dammed reservoirs with average depth of 10–30 m [21,22] and hydrodynamic properties partially similar to those of the deep water ecosystem. We defined these reservoirs as sub-deep reservoirs, differentiating them from typical deep reservoirs (with water depth > 30 m) for research purposes [22]. Sub-deep reservoirs are difficult to restore once polluted by excessive phosphorus, because they are too deep to be protected by macrophytes in their littoral zones and too shallow to mitigate phosphorus recycling through hypolimnetic dilution [15,23].

Over the last few decades, research efforts have been focused on the restoration of deep water system via reaeration [24,25,26,27,28,29,30]. Reaeration includes three main categories: water-mixing, hypolimnetic aeration and hypolimnetic oxygenation [14]. Among these, hypolimnetic oxygenation has received great attention for its higher restorative efficiency and fewer adverse effects [31]. Circular bubble plume diffusers (CBPD), one of the most popular technologies because of hypolimnetic oxygenation, consist of a gas source and a reaeration device and have been successfully operated in deep water systems [30,32], but have never been operated in sub-deep reservoirs.

It has been shown that CBPD can ensure high oxygen dissolution efficiency (90 ± 5%) in deep water systems with water depth beyond 30 m (even in Lake Hallwil with a depth of 46.5 m) [32,33]. However, this might be an arduous task in sub-deep reservoirs, because of the limited depth, probably resulting in an insufficient oxygen dissolution time and the bubble plume passing through the thermocline. Moreover, it remains unknown whether the bubble plume can break stratification. The effectiveness of CBPD in the sub-deep water system therefore remains questionable.

Oxygen introduced in water can be divided into three parts: oxygen escaped from water, oxygen dissolved in water, and oxygen consumed by oxygen-consuming matters. Previous works mainly focused on oxygen dissolved into water [34,35], while the other two parts were ignored. Investigation on the fate of the other two parts can significantly improve our understanding of the process of oxygenating the hypolimnion and the utilization efficiency of imported oxygen during hypolimnetic oxygenation.

In this study, we selected the Aha Reservoir, a typical sub-deep drinking water system in Southwest China, to carry out a field experiment to test CBPD in this system. The aims of this study were as follows: (1) to test the effectiveness of CBPD in a sub-deep water system; (2) to explore the consumption fate of imported oxygen during hypolimnetic oxygenation; (3) to reveal the intrusion characteristics of the circular bubble plume in the sub-deep water system during the period of thermal stratification; and (4) to determine whether CBPD is a feasible solution for restoring water quality in sub-deep reservoirs.

## 2. Materials and Methods 

### 2.1. Study Area

**Figure 1:**
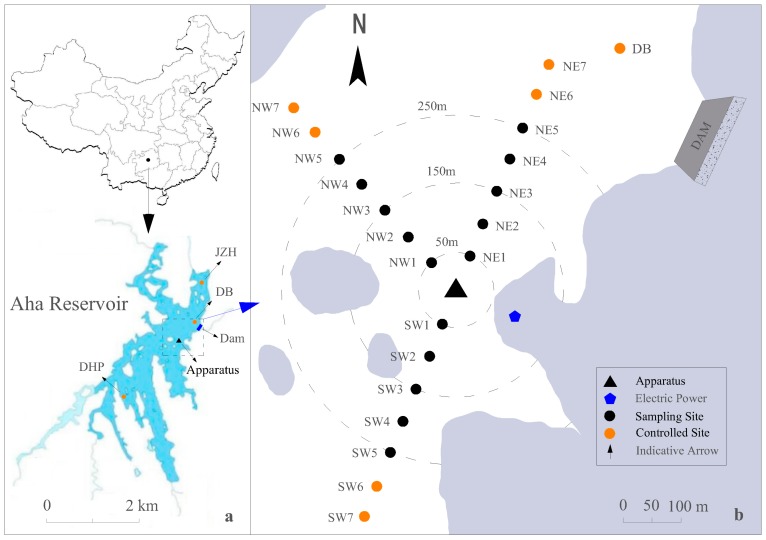
Study area (**a**) and sampling sites (**b**).

The Aha Reservoir (106°39′ E, 26°33′ N), constructed for the purpose of drinking water supply in 1960, is located in the suburbs of Guiyang, Southwest China (Figure 1a). It has a total surface area of 4.5 km^2^ and an average depth of 13 m (max. 26 m), with a watershed area of 190 km^2^ and a total volume of 4.45 × 10^7^ m^3^ at the normal water level of 1108 m [16]. The water residence time is 0.5 years and the water column is usually thermally stratified from late June to September [36,37]. During this period, serious oxygen deficit consistently occurs in the hypolimnion [38], resulting in the release of pollutants from sediment, the increase of primary productivity, and the aggravation of hypolimnetic anoxia [11,39]. The aggravation of anoxia in hypolimnion accelerates the release of pollutants from sediment, especially that of phosphorus, which induces annual algal blooms, and endangers the drinking water supply.

### 2.2. Reaeration Apparatus

**Figure 2:**
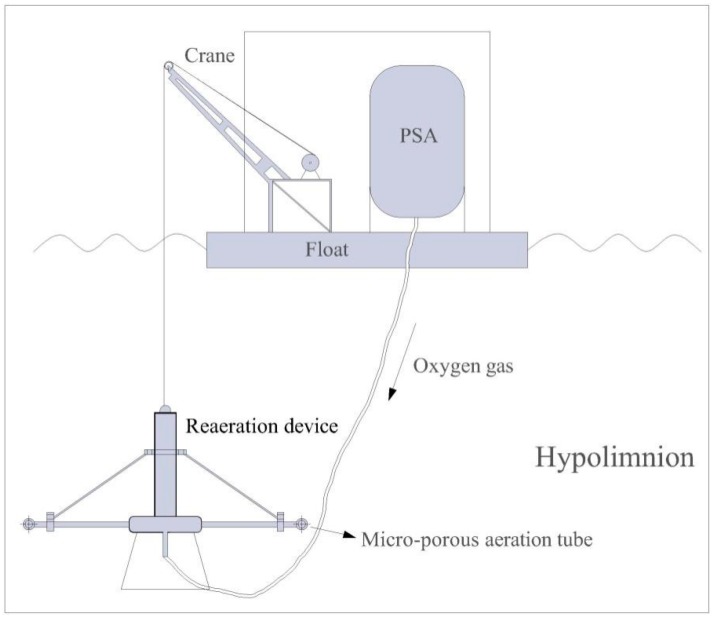
Sketch map of the reaeration apparatus.

The reaeration apparatus consists of a gas source and a reaeration device (Figure 2). Pure oxygen (94%) at a flow rate of 20 m^3^/h, was delivered to the reaeration device via a rubber hose by a Pressure Swing Adsorption system. The reaeration device was composed of a stainless steel frame and eight micro-porous tubes. Upon operation, tiny bubbles (jetted from thousands of orifices with diameter of 1 mm) were formed [30] and were injected into the water through a large number of dense micro-orifices attached to the tubes.

The reaeration apparatus was installed in a floating platform, 50 m away from the shore and 500 m away from the dam (Figure 1a). The average water depth was 20 m around the apparatus. The reaeration device was suspended 19 m below the surface and 2 m above the sediment.

### 2.3. Experimental Design

The biogeochemical cycling of manganese (Mn) is a complicated process and Mn is very difficult to chemically oxidize under natural conditions [40,41]. If oxygenation is stopped suddenly for a certain time (e.g., lasting for several weeks) after continuous oxygenation, Mn concentration of water body will rise rapidly [10,42]. We chose to operate our system at the beginning of the thermal stratification and throughout the whole period of summertime anoxia. The purpose of this operation is to prevent Mn concentration from rising in the hypolimnion. The reaeration apparatus was first operated for 12 h between 6:30 and 18:30 on 2 July 2016. Since then, the apparatus operated for 6 h between 12:30 and 18:30 each day from July to September. Water samples were collected from the upper, middle, and deeper layers at all sampling sites with a horizontal interval of 50 m (Figure 1b) every two weeks. Two control sites were set at the end of each direction, as well as DB, DHP and JZH. All the samples were stored at 4 °C before analysis. Water temperature (T) and DO were measured every 4 h during the daytime using a multi-parameter water quality monitoring instrument (YSI 6600V2, YSI Co., Ltd., Yellow Springs, OH, USA).

### 2.4. Water Chemistry Analysis

Total phosphorus (TP), total nitrogen (TN) and ammonia nitrogen (NH_4_^+^) were determined using the ammonium molybdate spectrophotometric method, alkaline potassium persulfate digestion-UV spectrophotometric method and Nessler’s reagent spectrophotometry, respectively. Inductively coupled plasma mass spectrometry (ICP-MS, NexION 300X, PerkinElmer Co., Ltd., Waltham, MA, USA) was used to determine the total iron (Fe) and total Mn in the water samples.

### 2.5. Oxygen Dissolution Efficiency Calculation

The oxygen dissolution efficiency (ODE, *η*) was determined using Equation (1) via the measurement of escaping bubbles. The integrated equations are as follows:

The dissolution efficiency of oxygen gas (*η*):

(1) $$\eta =\left[\left(Q_{0}-Q\right)/Q_{0}\right]\times 100\%,$$

where *Q* and *Q*_0_ represent volumetric flow rate of escaping bubbles (m^3^/h) and volumetric flow rate of initial bubble after pressure correction (m^3^/h).

The volumetric flow rate of initial bubble after pressure correction (*Q*_0_):

(2) $$Q_{0}=\left[\left(T_{0}\times Q_{1}\right)/T_{1}\right]\times \left(1+0.1\times H\right),$$

where *T*_0_, *T*_1_, *H*, and *Q*_1_ represent temperature in atmosphere (K), temperature at depth of reaeration device (K), depth of reaeration device (m) and volumetric flow rate of initial bubbles at the depth of reaeration device (m^3^/h), respectively.

The volumetric flow rate of escaping bubbles (*Q*):

(3) Q=F×S,

and the flux of escaping bubbles (*F*, m^3^/(h·m^2^)):

(4) $$F=\left(4\times V\right)/\left(\pi \times t\times d^{2}\right),$$

where *S* is the bubble plume area on water surface (m^2^); *V*, *t*, and *d* represent the volume of the bubble collection chamber (m^3^), time from starting collect bubbles to emptying the volume of the collection chamber (h), and the diameter of the inlet (m), respectively.

**Figure 3:**
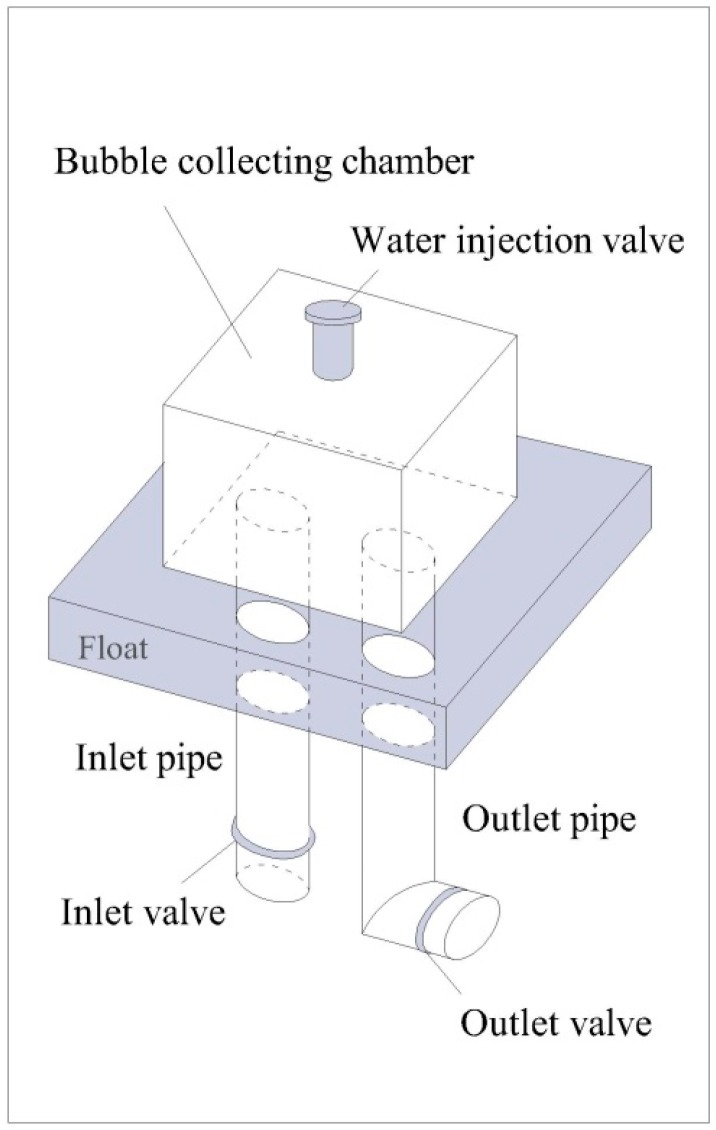
Diagram of the equipment for the measurement of oxygen dissolution efficiency.

Three valves were set on the equipment, including a water injection valve, an inlet valve and an outlet valve (Figure 3). The bubble collection chamber was full of saturated NaCl solution before measurement. The saturated NaCl solution was pushed out from the chamber as the room gradually filled with escaping bubbles. The collection time was recorded manually.

## 3. Results and Discussion 

### 3.1. Variations of DO within the Hypolimnion

**Figure 4:**
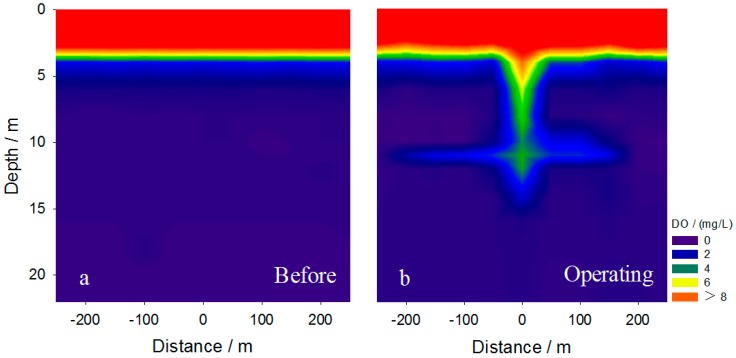
Spatial variation of dissolved oxygen (DO) in the water column (**a**) before and (**b**) during operation of the apparatus.

The ODE measurements indicate that the average ODE was as high as 95%, confirming that most oxygen was introduced into the aqueous phase. However, only a slight increase in concentration of DO was achieved within the hypolimnion, except in the intrusion layer (Figure 4). We postulate that most of the oxygen introduced into the water was consumed immediately by OM and other reduced substances (ORS, all reduced substances in water) in the hypolimnion, such as reduced Fe, Mn, sulfur (S^2−^) and NH_4_^+^, resulting in the limited oxygenation effect. This is supported by the significant decline in concentrations of NH_4_^+^, total Fe and total Mn in the experimental zone (Table 1), with net decreasing percentages of 15.5%, 45.5% and 48.9% compared with the control sites, respectively.

### 3.2. Mass Balance of Oxygen

Oxygen introduced in water can be divided into three components: oxygen escaped from water, oxygen dissolved in water, and oxygen consumed by oxygen-consuming matter. Previous studies associated with reaeration seldom systematically studied the fate of imported oxygen during hypolimnetic oxygenation. A mass balance equation was introduced to clearly describe the consumption fate of oxygen imported into water:

(5) $$n_{i}=n_{DO}+n_{e}+n_{1}+n_{2},$$

where *n_i_*, *n_DO_*, and *n_e_* represent the total imported oxygen, oxygen dissolved in water, and oxygen escaped from water, respectively; *n*_1_ represents the oxygen consumed by NH_4_^+^, Fe and Mn; *n*_2_ represents the oxygen consumed by OM and the remaining reduced materials (RRM) of ORS (excluding NH_4_^+^, Fe, and Mn, and including mainly S^2−^, nitrite (NO_2_^−^), and methane (CH_4_)). The calculated results indicated that *n_DO_* and *n_e_* represented only 4.2% and 5% of the total imported O_2_, and up to 90.8% of the total imported O_2_ was involved in the consumption processes. The areal hypolimnetic mineralization (AHM) rate (g O_2_/m^2^/day) [39] is an integrated index on the potential of oxygen depletion in hypolimnion and can be calculated with the following equation [43]:

(6) $$AHM=F_{red}+\left[D_{O_{2}}/\left(\delta \times \Delta t\right)\right]\;\int_{for\;C>0}^{0-\Delta t}\;C\;\left(t\right)\;dt,$$

where *F_red_* is the diffusive benthic flux of reduced substances diffusing into the water across the specific area (g O_2_/m^2^/day), *D_O_*_2_ is the diffusion coefficient of molecular O_2_ (m^2^/day), *δ* is the thickness of diffusive boundary layer (m), Δ*t* is the time of lake stratification period (days), and *C*(*t*) is the O_2_ concentration (mg/L). As oxygen can only be consumed as long as it is available, the integration was applied only during periods in which DO > 0 mg/L.

The calculated AHM rate in the Aha Reservoir is 0.8 g O_2_/m^2^/day, which is comparable to the predicted result of 0.6 g O_2_/m^2^/day for a mean hypolimnion depth of 6 m [43]. The difference between these values may be attributed to the difference in hypolimnetic conditions.

There are several ways to consume the replenished DO in the hypolimnion and the consumption of oxygen can be calculated based on the following equations:

(7) $${\mathrm{NH}}_{4}^{+}+{2O}_{2}↔{\mathrm{NO}}_{3}^{-}+H_{2}O+{2H}^{+}$$

(8) $${\mathrm{Fe}}^{2+}+0.25O_{2}+H^{+}↔{\mathrm{Fe}}^{3+}+0.5H_{2}O$$

(9) $${\mathrm{Mn}}^{2+}+0.5O_{2}+{2H}^{+}↔{\mathrm{Mn}}^{4+}+H_{2}O$$

The results show that the consumption of oxygen for NH_4_^+^, Fe and Mn was 0.046, 1 × 10^−4^ and 1.8 × 10^−5^ mol/m^3^, respectively. The consumption of oxygen via the oxygenation of Fe and Mn was extremely limited. Thus, *n*_1_ was primarily consumed by NH_4_^+^, accounting for 25% of the total imported O_2_. Potential mechanisms for the depletion of NH_4_^+^ in the presence of oxygen include: (1) restraining the release of NH_4_^+^ from the sediments during hypolimnetic oxygenation, and (2) oxidation of NH_4_^+^ followed by escaped into the atmosphere [44].

Apart from the fates of oxygen mentioned above, 65.8% of the total imported oxygen remained, which was probably consumed by the other reduced substances, such as S^2−^, NO_2_^−^, CH_4_ and OM as follows:

(10) $$S^{2-}+{2O}_{2}↔{\mathrm{SO}}_{4}^{2-}$$

(11) $${\mathrm{NO}}_{2}^{-}+0.5O_{2}↔{\mathrm{NO}}_{3}^{-}$$

(12) $${\mathrm{CH}}_{4}+{2O}_{2}↔{\mathrm{CO}}_{2}+{2H}_{2}O$$

(13) $${\left({\mathrm{CH}}_{2}O\right)}_{106}\;{\left({\mathrm{NH}}_{3}\right)}_{16}\;\left(H_{3}{\mathrm{PO}}_{4}\right)+{138\;O}_{2}↔{106\;\mathrm{CO}}_{2}+{16\;\mathrm{HNO}}_{3}+H_{3}{\mathrm{PO}}_{4}+{122\;H}_{2}O$$

A large amount of algae OM has been deposited in the Aha Reservoir from algal blooms, which is easily degraded under aerobic conditions [45]. Based on Equations (10)–(13), the OM has a much greater oxygen consumption capacity than S^2−^, NO_2_^−^ and CH_4_. Fluxes of oxygen consumption by reduced substances are estimated in the range of 0.34–0.44 g O_2_/m^2^/day [39], Thus, the oxygen consumption of OM and RRM, accounted for 41.4–52.5% and 13.3–24.4% of imported oxygen, respectively, indicating that the introduced oxygen was primarily consumed by OM and ORS.

### 3.3. Effect on the Stability of Thermal Stratification

The temperature gradient is an important feature of the thermocline. Clear seasonal stratification occurs in the Aha Reservoir in summer [37]. Maintaining the stability of thermal stratification is crucial to protect the living conditions for benthic organisms [33]. During the experiment, we found that the horizontal intrusive gravity currents reached far-field and mainly happened within the depth 10–14 m, an equilibrium depth close to the thermocline within the depth of 9–14 m (Figure 4). This indicates that the horizontal intrusion mainly occurred within the thermocline during the stratification and raised concerns on whether the tiny bubbles and the intrusive plumes destroyed the thermal stratification when they vertically passed through or horizontally invaded the thermocline.

**Figure 5:**
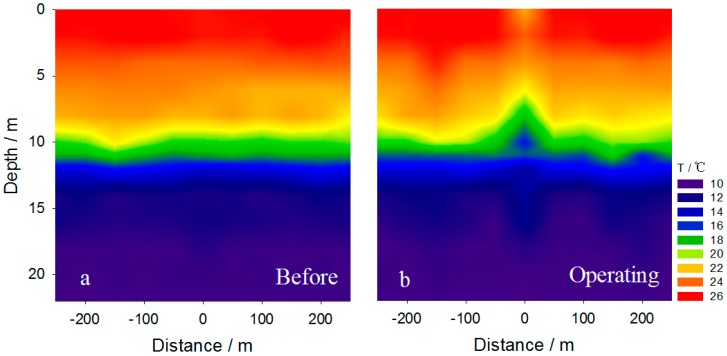
Spatial variation of temperature in the water column (**a**) before and (**b**) during operation of the apparatus.

**Figure 6:**
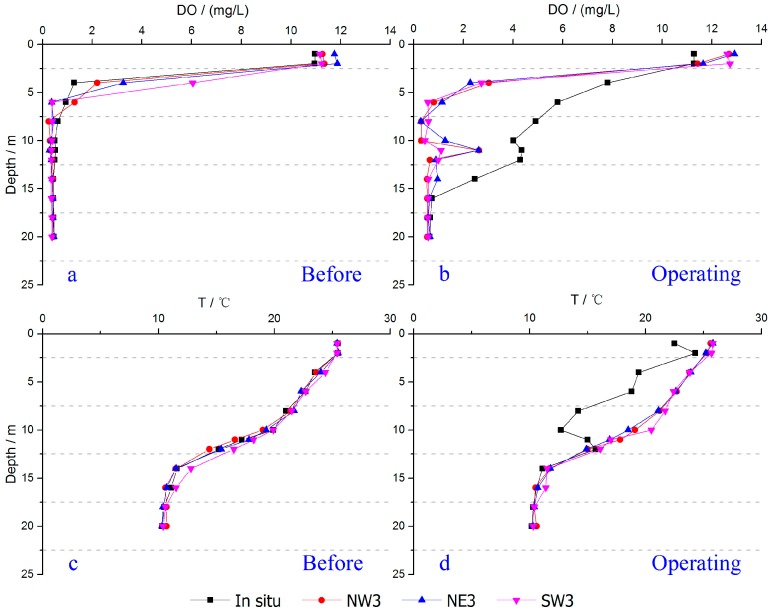
Variations of DO (**a**) before and (**b**) during operation of the apparatus, and T (**c**) before and (**d**) during operation of the apparatus in the water column during stratification.

As illustrated in Figure 5a, there was a stable thermocline with a clear temperature gradient within the depth of 9–14 m prior to the experiment. During operation, the temperature of benthic water was the same as before and the thermocline remained in place (Figure 5b). This indicates that the hypolimnetic oxygenation had no significant influence on the thermocline, though a slight disturbance was observed (Figure 6b). Detailed measurements suggested that the water temperature below the depth of 12 m remained as before, indicating that the thermocline was present. This is also supported by the same vertical profiles of temperature at Sites NW3, NE3 and SW3 before and after operation (Figure 6c,d). However, because of the uplift of cold benthic water with the bubble plume, water temperature in situ decreased distinctly within the depth of 0–12 m (Figure 6d). This was probably the main cause of the slight disturbance of the thermocline observed in the central area (Figure 5b).

### 3.4. Intrusion Characteristics

We found that 95% of the tiny bubbles were dissolved in water before they reached the surface as mentioned above. In addition, intrusion was successfully achieved as illustrated in Figure 4. Vertically, a significant increase in DO level within the 4–16 m depth was observed in the central area and a roughly axisymmetric intrusion of oxygen-enriched water occurred at 10–14 m below the surface (Figure 4 and Figure 6b). Horizontally, the axisymmetric intrusive gravity currents at equilibrium depth reached as far as Site NW5, 250 m from the central area (Figure 4), indicating a radius of intrusion of approximately 250 m. This demonstrates the effectiveness of CBPD in the Aha Reservoir.

### 3.5. Effect on the Water Quality

Phosphorus and nitrogen are the primary nutrient elements associated with eutrophication in aquatic ecosystems [46,47,48] and have become the major pollutants in the Aha Reservoir [49]. Hypolimnetic oxygenation can logically result in elevated DO in benthic water and restrain the release of nutrient elements from sediment, which is favorable for water quality.

The formation of thermal stratification limits the exchange between upper water and deep water, resulting in the anoxia in hypolimnion, and thereby, promoting the release of nutrients from sediment. In 2015, most concentrations of TP and TN increased gradually in the vertical profile from surface to bottom. As shown in Table 2, within the studied area, the average concentrations of TP were 0.04, 0.02 and 0.02 mg/L in July, August and September of 2016, respectively. The corresponding data on TN were 1.9, 1.7 and 1.6 mg/L, respectively. They were significantly lower than the corresponding values collected in 2015 (before CBPD operating).

**Figure 7:**
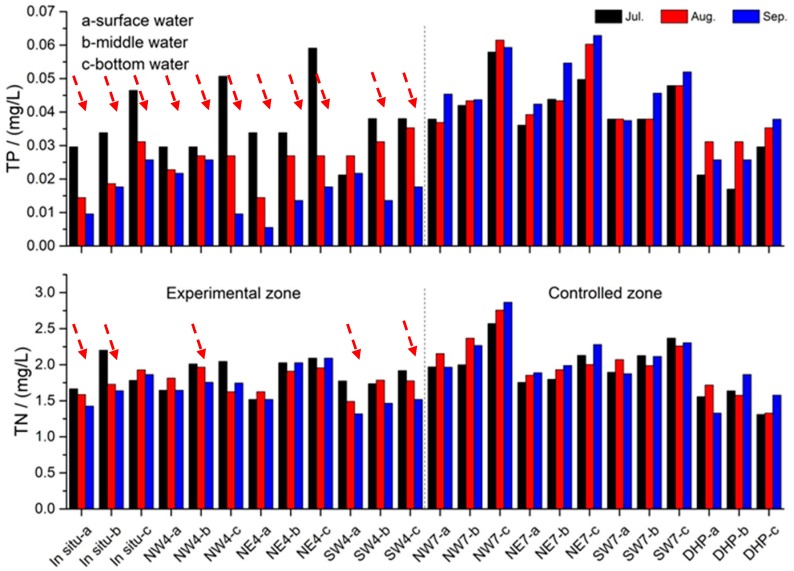
Variations of total phosphorus (TP) and total nitrogen (TN) in the experimental zone and the control zone.

Concentrations of TP and TN were compared in the experimental zone and control sites (Figure 7). Student’s *t*-test was used to analyze the difference of water quality during oxygenation (Table 3). The results indicated that there was a significant decrease in TP and TN concentrations in the experimental zone (0.04–0.02 mg/L and 1.9–1.7 mg/L, respectively), with no significant decrease in TP and TN concentrations in the control sites. This demonstrates that CBPD was effective in reducing TP and TN in the Aha Reservoir. Two mechanisms were responsible for the decrease in total phosphorus in water. Firstly, the CBPD system can greatly inhibit the release of phosphorus from sediment via increasing the dissolved oxygen concentration in the bottom water. A large body of evidence has confirmed that phosphorus in sediment is more readily released under anaerobic conditions than aerobic conditions, especially BD-P [11,50]. Secondly, elevated dissolved oxygen concentration in the water body may promote the precipitation of phosphorus from water [8]. As for the decrease in total nitrogen, the mechanism may be more complicated. This is also likely to be the result of the increase of DO. Previous investigation has confirmed that nitrogen can be released from sediment in the form of ammonia [51], due to a loss of biological nitrification and a decrease in ammonia assimilation by anaerobic microorganisms under anaerobic condition [52]. CBPD system can inhibit the release of ammonia from sediment via increasing DO in the water body. Meanwhile, the release of nitrate is still limited, because most of the resulting nitrate can be lost from the aquatic ecosystem via denitrification in subsurface sediment or anoxic micro-zones [52]. No clear decrease in nutrients was observed at some sampling sites. This was probably because the experimental area was not isolated from the surroundings.

The CBPD application is helpful to the improvement of drinking water quality. During the past few years, algal blooms occurred every summer in the Aha Reservoir [16,37]. After the application of CBPD in 2016, algal abundance in the studied area decreased obviously [53] and no algal bloom was observed till July 2017. Three short-term benefits to drinking water from the CBPD application include: (1) DO in the hypolimnion increased 3–4 times and concentrations of Fe and Mn were reduced substantially (45.5% and 48.9%); (2) the average concentrations of TP and TN in the studied area decreased significantly after CBPD operation (Figure 7); and (3) algae abundance was greatly reduced from 1.07 × 10^8^ to 2 × 10^6^ cells/L, and the percentage of cyanobacteria decreased simultaneously from 96.3% to 25% [53].

## 4. Conclusions

In this study, CBPD was applied for the first time to restore water quality in the Aha Reservoir, a typical sub-deep reservoir in Southwestern China. The spatial variations in DO demonstrated the formation of axisymmetric intrusive gravity currents. Compared with the control sites, the DO level in hypolimnion increased by 3–4 times, and the concentrations of TP, TN, NH_4_^+^, Fe, and Mn decreased significantly in the experimental zone. This indicates that CBPD has great potential for oxygenation of the hypolimnion and improving water quality in the sub-deep water system.

The injected oxygen gas was primarily consumed by OM and ORS within the hypolimnion, which has not been considered fully in the discrete bubble models that are widely used at present. Further efforts are needed to improve the discrete bubble model to elaborate the oxygen transmission dynamics and the plume formation processes in the sub-deep water system, with incorporation of oxygen consumption processes.

## Figures and Tables

**Table 1:** Concentrations of NH_4_^+^ (mg/L), total Fe and total Mn (µg/L) at the sampling sites and the percentage net decrease of NH_4_^+^, total Fe and total Mn (PND, %) for the corresponding sites from July to September.

Sites	July			September			PND		
	NH_4_^+^	Total Fe	Total Mn	NH_4_^+^	Total Fe	Total Mn	NH_4_^+^	Total Fe	Total Mn
In situ	0.48–0.71	37.6–67.5	2.4–12.0	0.16–0.34	4.40–91.4	0.4–1.4	30–71	74–94	74–88
NW4	0.47–0.54	8.10–45.4	0.8–1.5	0.08–0.27	0.10–13.4	0.2–0.4	49–84	94–99	53–86
NE4	0.47–0.57	5.10–45.7	0.6–1.3	0.14–0.32	0.40–33.5	0.1–0.8	43–71	27–92	36–81
SW4	0.56–0.59	11.3–42.4	1.1–4.6	0.14–0.32	3.30–10.8	0.3–0.5	46–76	79–91	74–88
DB	0.34–0.35	15.7–27.4	1.4–2.2	0.18–0.22	13.6–58.3	1.1–1.8	38–47	51–60	17–21
JZH/DHP *	0.42–0.45	23.3–29.7	1.8–2.1	0.14–0.34	11.4–33.0	1.0–1.2	19–69	10–51	35–49

DHP * represents the concentration and PND of NH_4_^+^ in the Site DHP.

**Table 2:** Comparison of TP and TN concentrations (mg/L) in the studied area between 2015 and 2016.

	Jul. 2015	Aug. 2015	Sep. 2015	Jul. 2016	Aug. 2016	Sep. 2016
**TP** (mg/L)						
Upper	0.03	0.05	0.04	0.03	0.01	0.01
Middle	0.03	0.06	0.05	0.03	0.02	0.02
Bottom	0.06	0.07	0.05	0.05	0.03	0.03
AVG	0.04	0.06	0.05	0.04	0.02	0.02
**TN** (mg/L)						
Upper	1.8	2.3	2.1	1.7	1.6	1.4
Middle	2.0	2.8	2.3	2.2	1.7	1.6
Bottom	2.1	3.0	2.1	1.8	1.9	1.9
AVG	2.0	2.7	2.2	1.9	1.7	1.6

**Table 3:** Student’s *t*-test results for TP and TN in water (a = 0.05).

	Jul.	Aug.	Sep.	Aug. & Sep.	Aug. & Sep.	Jul.	Jul. & Aug.		Aug. & Sep.		Jul. & Sep.		Jul.		Aug. & Sep.	
	E.Z.	E.Z.	E.Z.	E.Z.	C.S.	C.S.	E.Z.		E.Z.		E.Z.		E.Z. & C.S.		E.Z. & C.S.	
**TP**	$\bar{x}$	$\bar{x}$	$\bar{x}$	$\bar{x}$	$\bar{x}$	$\bar{x}$	|*t*|	*p*	|*t*|	*p*	|*t*|	*p*	|*t*|	*p*	|*t*|	*p*
	0.04	0.03	0.02	0.02	0.04	0.04	3.3	0.003	3.2	0.004	5.7	9 × 10^‒6^	0.28	0.8	8.3	1 × 10^‒10^
**TN**	$\bar{x}$	$\bar{x}$	$\bar{x}$	$\bar{x}$	$\bar{x}$	$\bar{x}$	|*t*|	*p*	|*t*|	*p*	|*t*|	*p*	|*t*|	*p*	|*t*|	*p*
	1.9	1.8	1.7	1.7	2.0	1.9	1.3	0.2	1.2	0.3	2.2	0.04	0.49	0.6	3.4	0.001

$\bar{x}$ represents the average value (mg/L), |*t*| represents the absolute value of the *t*-statistic, and *p* represents the probability value, respectively. E.Z. represents the experimental zone, C.S. represents the control sites. a = 0.05 represents the significance level.
